# The AACR 2013 in Washington D.C.

**DOI:** 10.3332/ecancer.2013.ed19

**Published:** 2013-05-17

**Authors:** Giovanni Codacci-Pisanelli

**Affiliations:** Universita de Roma 1, Rome, Italy

The annual meeting of the American Association for Cancer Research (AACR) is always a major event: the presence of 18,000 people from all over the world is something amazing! And even more so when one considers the young age of participants and of speakers (in comparison with other conferences). The presence of different sessions going on at the same time often requires difficult choices, and an athletic attitude to keep running from one side of the conference venue to the other!

A peculiarity of the AACR meeting is that it includes every field of oncology, from carcinogenesis to early clinical trials. This contrasts with the tendency to organise conferences on a specific topic, and it brings together scientists that otherwise would have little or no occasion of meeting. This non-specificity represents a distinguishing trait from ASCO, the clinical relative of AACR, and reduces the overlap between the two conferences. One attitude when coming to the AACR is to try to spot the subjects that will be discussed at ASCO in the following years: I am not sure that this is acceptable even if it certainly acknowledges the translational spirit of cancer research.

A recent paper questioned the use of medical meetings [1] and certainly the fast publication of results in electronic form, with the frequent appearance of “early releases”, allows a fast spread of results. In this sense meetings have partly lost their importance as the first place to present early data: but a conference is not only a place where you come to know new data. Here you have the possibility of interacting with your colleagues (friends and competitors): talking to them and discussing in person, something very different from a Skype call!

This is why the sessions I prefer are posters! You can stroll along the endless lines of panels and stop when a title catches you eye, or you can walk straight to a poster you spotted on the programme: in both cases you can discuss with the presenter until one of you is physically exhausted!

At the same time I admire “informal meetings”: circles of people sitting around a table or (more often) on the floor, possibly close to an electricity plug and looking at the screen of a portable computer. They listen and discuss passionately, almost unaware of the hundreds of people walking the same corridor towards a presentations on a dozen of different subjects.

From a distance the conference centre appears as a mass of moving points speeding in all directions, and the presence of escalators creates a three-dimensional chaos! This loose swarm will then be compacted into huge crowded rooms and then silence reigns during the presentation. At the end a round of applause…and the corridors are once again full of people hurrying to the next session!

This ebb and flow goes on from the early morning, with sessions starting before seven o’clock, until late afternoon. It is during these unstoppable movements that you may notice the most famous names in cancer research or a friend speeding in the opposite direction.

The 2013 conference did not have a definite “hot topic” but among the most widely discussed subjects were RNAs, epigenetics, “liquid biopsies”, the link between cancer and metabolism/inflammation, the application of genetics to immunological treatments.

For many years RNA was only considered as an ancillary molecule, but today different roles are attributed to this molecule. Paradoxically what now receives most attention is the portion or RNA that is not coding for genes. Non-coding sequences play essential roles in the regulation of gene expression and micro-RNAs, that also have similar activities, receive special attention since they are being used for treatment.

The regulation of gene expression has been traditionally ascribed to the epigenetic mechanisms of DNA methylation and of histone methylation and acetylation/deacetylation. Since the identification of new and old drugs that interfere with the epigenetic regulation of gene expression this field in rapidly expanding and partly merges with the study of regulatory RNA molecules.

Liquid biopsies refer to the analysis of tumour material that is released in plasma: it started with the analysis of circulating tumour cells (CTC) but now also includes the analysis of DNA and of microRNAs. These techniques are particularly relevant when a tumour biopsy cannot be easily obtained and when it is necessary to monitor the molecular response to treatment. The intention is to identify molecular types of tumours similar to what has been obtained for breast cancer and to identify the single patient that will benefit from a specific treatment by evaluating the relevance of the molecular target in the tumour. This also applies to the changes associated with the development of resistance and the identification of different targets for second line treatment.

The relationship between cancer and inflammation has been extensively studied since the times of Warburg, but this subject has now acquired new impetus since the role of metabolism in these processes has been recognised.

Genetic analysis for immunological treatment: this subject recently appeared on stage. The success of immune manipulation in melanoma certainly was a strong impulse, but now it also includes the treatment of leukaemia using genetically modified immunological cells.

Great emphasis was given to the Stand up to Cancer (SU2C) initiative, and it certainly deserves it! The AACR coordinated this initiative and funded with generous amounts of money different research lines that brought together different research groups with distinct expertise and transferred results from basic research to the patients at an exceptional speed.

Washington with its wonderful weather and the charm of the cherry blossom contributed to make this conference a very pleasant experience, next year it will be in San Diego, and we look forward to enjoying the annual AACR meeting once more!
